# Functional and expression analyses of transcripts based on full-length cDNAs of *Sorghum bicolor*

**DOI:** 10.1093/dnares/dsv030

**Published:** 2015-11-05

**Authors:** Setsuko Shimada, Yuko Makita, Tomoko Kuriyama-Kondou, Mika Kawashima, Yoshiki Mochizuki, Hideki Hirakawa, Shusei Sato, Tetsuro Toyoda, Minami Matsui

**Affiliations:** 1 Synthetic Genomics Research Group, Biomass Engineering Research Division, RIKEN Center for Sustainable Resource Science, Yokohama, Kanagawa 230-0045, Japan; 2 RIKEN Advanced Center for Computing and Communication (ACCC), Wako, Saitama 351-0198, Japan; 3 Kazusa DNA Research Institute, Kisarazu, Chiba 292-0818, Japan

**Keywords:** *Sorghum bicolor*, full-length cDNA, RNA-Seq, transcriptome

## Abstract

*Sorghum bicolor* is one of the most important crops for food and bioethanol production. Its small diploid genome and resistance to environmental stress make sorghum an attractive model for studying the functional genomics of the Saccharinae and other C_4_ grasses. We analyzed the domain-based functional annotation of the cDNAs using the gene ontology (GO) categories for molecular function to characterize all the genes cloned in the full-length cDNA library of sorghum. The sorghum cDNA library successfully captured a wide range of cDNA-encoded proteins with various functions. To characterize the protein function of newly identified cDNAs, a search of their deduced domains and comparative analyses in the *Oryza sativa* and *Zea mays* genomes were carried out. Furthermore, genes on the sense strand corresponding to antisense transcripts were classified based on the GO of molecular function. To add more information about these genes, we have analyzed the expression profiles using RNA-Seq of three tissues (spikelet, seed and stem) during the starch-filling phase. We performed functional analysis of tissue-specific genes and expression analysis of genes of starch biosynthesis enzymes. This functional analysis of sorghum full-length cDNAs and the transcriptome information will facilitate further analysis of the Saccharinae and grass families.

## 1. Introduction

Sorghum [*Sorghum bicolor* (L.) Moench] is the most important staple cereal crop for millions of people living in West Africa and India,^1^ providing food for human consumption and feed grain for livestock. More recently, it has become the focus for industrial applications such as bioethanol production. Not only it is an important cereal crop, but also its small diploid genome (∼730 Mb) makes sorghum an attractive model for studying functional genomics of the Saccharinae and other C_4_ grasses. Sorghum belongs to the Saccharinae subfamily, which includes some of the most efficient biomass accumulators, such as sugarcane (*Saccharum officinarum*) and *Miscanthus* (*Miscanthus giganteus*)*.* They use C_4_ photosynthesis, but there is morphological and genomic variations.^2^ Sorghum is closely related to sugarcane, maize (*Zea mays*) and switchgrass (*Panicum virgatum*), which have much larger polyploid genomes.^3^ Sorghum genomic information is useful for further analysis of these plants. In addition, sorghum is a good model for studying plant response to abiotic stress, particularly to drought and high temperature stresses.^4^ Sorghum's tolerance to such stresses makes it especially important among other grasses. Researching sorghum's genome is valuable to accelerate the discovery of new genes and to understand the function and regulation of genes underlying agronomic or compositional traits. For these reasons, the genome sequence of grain sorghum BTx623 was determined in 2009 by the whole-genome shotgun sequencing method.^2^ The development of genomic resources and comparative genomics of sorghum will accelerate functional analysis of genes and specific properties of grass species.

In addition to entire genomic sequences, collections of expressed sequence tags (ESTs) have been made to improve knowledge of the sorghum transcriptome. A total of 117,682 ESTs have been obtained from sorghum cDNA libraries prepared from samples under various conditions.^5^ There are currently over 2 million ESTs of sorghum in Genbank.^6^ EST data are a valuable resource for making expression catalogues. A partial sequence of a transcript is not reliable for correct gene annotation in the genome sequence or for prediction of the coded protein. Full-length cDNAs contain complete coding sequences as well as the 5′ and 3′ untranslated regions. Consequently, genome-scale collections of full-length cDNAs are important for further analysis of the structure and function of genes and their coding proteins.

Over the last decade, microarray-based expression profiling experiments for genome-wide analysis in sorghum have been conducted to examine responses to various abiotic and biotic stresses,^7–10^ to identify tissue-specific and genotype-specific gene expression patterns,^11^ and to reveal genetic variation and expression diversity between grain sorghum, BTx623, and sweet sorghum, Keller.^12^ Grain sorghum is important cereal crop for food and feed, while sweet sorghum has a high biomass yield and sugar content. Both lines originated from the same species, *S. bicolor*. Furthermore, next-generation sequencing technology has provided a more complete view of gene expression and their networks in sorghum. RNA-Seq technology for expression analysis has been employed in sorghum to examine its response to osmotic stress, abscisic acid (ABA)^13^ and pathogens,^14,15^ to identify genes responsible for low nitrogen tolerance^16^ and to compare analogous transcriptomes with other Poaceae plants.^17^ The advantage of RNA-Seq is that it provides an accurate assessment of gene expression at various stages and under different conditions, which will allow a more detailed atlas of gene expression in sorghum to be produced and thus enable functional analysis of its genes.

We have reported two approaches to accelerate progress in sorghum genomics.^18^ One was a large-scale collection of full-length cDNAs from *S. bicolor* BTx623 for further accurate annotation of the sorghum genome, e.g. proper understanding of transcription start sites. We have collected ∼40,000 full-length cDNAs and identified new genes and antisense transcripts. The other approach was a genome-wide transcription analysis using RNA-Seq to add information about the expression profiles of our cloned full-length cDNAs. We have reported analysis of the expression profiles of three tissues, the spikelet, the seed and the stem. Furthermore, we have established a web-accessible database, MOROKOSHI (http://sorghum.riken.jp).^18^ In this database, full-length cDNAs and our original RNA-Seq data, as well as publicly available sorghum RNA-Seq data, can be shown on a genome browser together.

In this study, we performed functional analysis of genes cloned in our full-length cDNA library, including the newly identified genes and antisense transcripts. Furthermore, we performed functional analysis of tissue-specific genes and expression analysis of genes of enzymes involved in starch biosynthesis. This functional analysis of a large-scale collection of full-length cDNAs with genomic and transcriptome information will facilitate the location of functional genes in the Saccharinae and grass families.

## 2. Materials and methods

### 2.1. Functional analysis

The full-length cDNA data of 10,811-non-redundant genes cloned in the normalized sorghum full-length cDNA library^18^ was used for functional analysis. The library was made from total RNA of soil-grown *S. bicolor* BTx623 from various aerial tissues including stems, leaves, spikelets and seeds, and at different developmental stages.^18^

The NCBI CDD (conserved domains database) was used to identify functional domains in identified genes.^19^

For sorghum gene ontology (GO) annotation, we used agriGO,^20^ a GO analysis toolkit for the agricultural research community. To summarize GO functional annotations, we used the GOSlimViewer on the AgBase^21^ website.

### 2.2. RNA-Seq preparation

RNA-Seq libraries were prepared from total RNA of each tissues, spikelet, seed and stem using Illumina TruSeq Stranded mRNA Library Preparation Kits (Illumina, San Diego, CA, USA), according to the manufacturer's protocol.^18^ We performed directional RNA-Seq with a HiSeq2000 (Illumina). The read length was 50 bp of single reads and sequence read data were submitted to the DDBJ (PRJDB3281).

### 2.3. Analysis of RNA-Seq data

For sequence quality control, we used the FASTX-Toolkit (http://hannonlab.cshl.edu/fastx_toolkit/). First, we trimmed base pairs with a Phred quality value (QV) of <20 from the 3′ end of each sequence and discarded the sequence when it was shorter than 30 bp in length. Next, if >20% of a sequence had a QV of <20 then it was discarded. Sequences that passed these two filters were mapped with TopHat v2.0.11, assembled and compared using Cufflinks v2.2.0.

For pathway enrichment analysis, the gene-pathway annotations were derived from KEGG.^22^ We applied a hypergeometric test to identify significantly enriched pathways:

$$P\text{-value}=1-\sum_{i=0}^{m}\frac{\left(\begin{array}{c} M\\ i \end{array}\right)\left(\begin{array}{c} N-M\\ n-i \end{array}\right)}{\left(\begin{array}{c} N\\ n \end{array}\right)},$$

where *N* is the total number of all genes with KEGG pathway annotation, *n* is the number of differentially expressed genes (DEGs) in N, M is the number of genes in a given pathway, and m is the number of DEGs in M. We then controlled the proportion of false positives by calculating the false discovery rate (FDR) corresponding to each *P*-value. The pathways with a *q*-value of ≤0.05 were defined as significantly enriched genes in 3 way pairwise comparisons.

## 3. Results and discussion

### 3.1. Functional analysis of sorghum genes in association with full-length cDNAs

We have reported the construction of a normalized *S. bicolor* full-length cDNA library from BTx623 using the biotinylated CAP-trapper method with trehalose-thermoactivated reverse transcriptase^23–25^ to facilitate the discovery of novel transcription units and to enable accurate structural gene annotation.^18^

**Figure 1.**
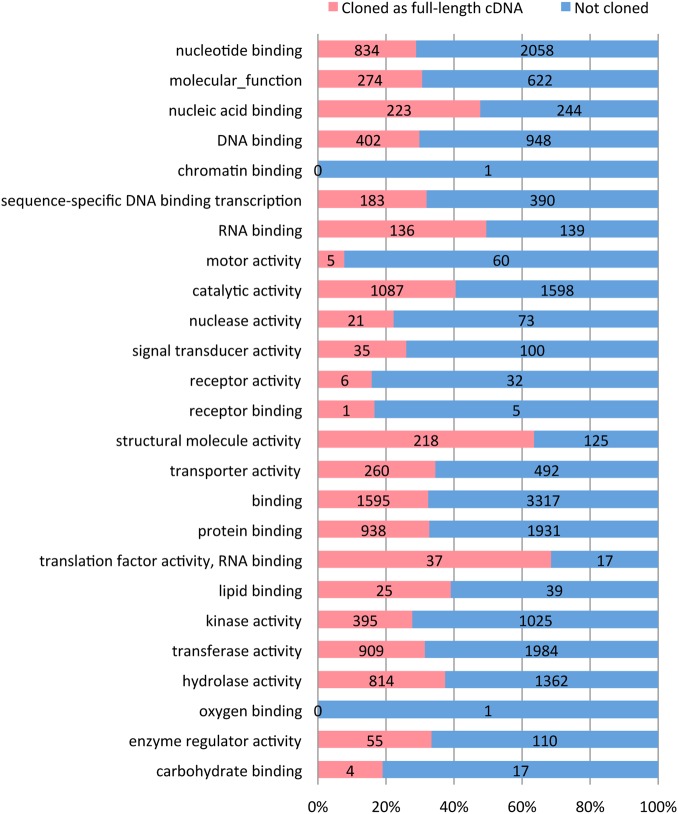
Ratio of sorghum genes cloned as full-length cDNAs in a functional classification based on GO slim categories of available genes annotated in sorghum genome (Sbicolor_255). The represented data is for the category of molecular function. The labels show the number of genes.

To characterize all the genes cloned in the full-length cDNA library, we analyzed the domain-based functional annotation of the cDNAs. The functional motifs or domains were identified from protein predictions and classified using the GO categories for molecular function. GO analysis of genes cloned in the library and other genes annotated in the sorghum genome (Sbicolor_255) reveals that the categories of ‘structural molecule activity’ and ‘translation factor activity, RNA binding’ had high coverage, whereas the categories of ‘chromatin binding’, ‘motor activity’, ‘receptor activity’ and ‘receptor binding’ showed low coverage. Other categories had ∼30% coverage (Fig. 1). The sorghum cDNA library successfully captured a wide range of cDNA-encoded proteins with various functions. Furthermore, to confirm whether our full-length cDNAs contain genes expressed in specific conditions, a comparative analysis using genes regulated by osmotic stress and ABA treatment was performed. The transcriptomes of sorghum in response to osmotic stress and ABA have been reported.^13^ They showed 5,156 DEGs under polyethylene glycol (PEG) or ABA treatment. We used 5,141 DEGs, having eliminated miRNAs, from a total of 5,156 DEGs for the comparative analysis. The results showed that 31.6% genes (1,623 genes out of 5,141 genes) were present in the sorghum full-length cDNA library (Supplementary Fig. S1). This indicates that the library partially captured genes expressed in specific conditions.

**Figure 2.**
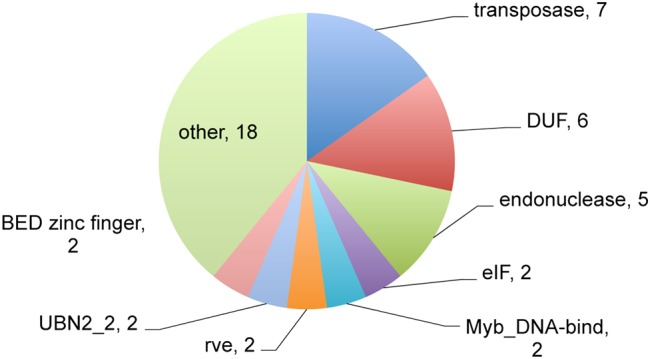
Domain search of products from newly identified genes. The pie chart represents the number of genes predicted to include encode the different domains. Newly identified genes were classified based on the domains predicted using the NCBI CDD (conserved domains database; http://www.ncbi.nlm.nih.gov/cdd). DUF, domain of unknown function; eIF, eukaryotic translation initiation factor; rev; integrase core domain; UBN, gag-polypeptide of LTR copia-type.

To characterize the protein function of newly identified cDNAs, a search of their deduced domains was carried out (Fig. 2). In our full-length cDNA library, we have determined 336 newly identified genes.^18^ The domains are distributed in most of the criteria with an abundance of transposase domains. A total of 40 (14.7%) newly identified genes contained domains. Myb domains were also present, which suggests some newly identified genes act as transcription factors. A protein kinase domain and a F-box domain were also found. All this suggests that the newly identified genes encode proteins with various functions.

**Figure 3.**
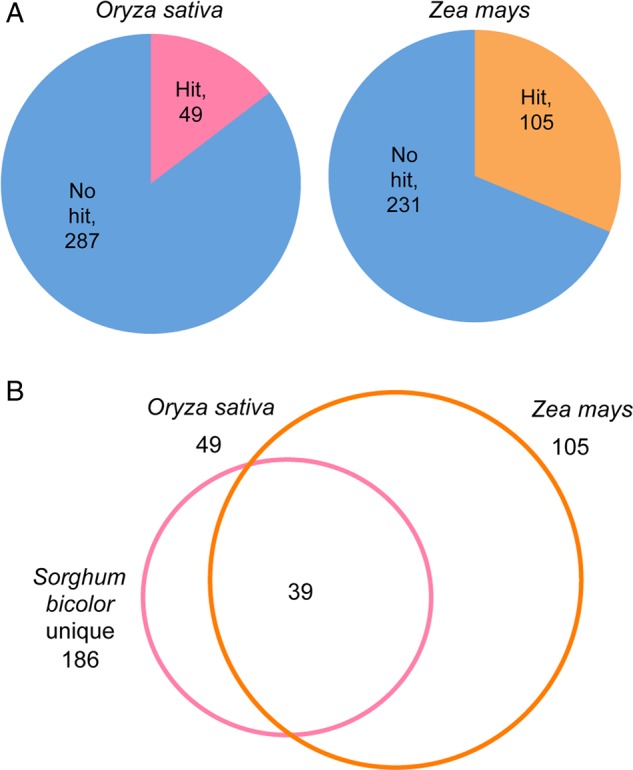
Comparative analyses of newly identified genes in *O. sativa* and *Z. mays* genomes. The newly identified genes were searched against annotated genes of each species. (A) Pie chart shows the number of genes that were hit or were not hit in each species. (B) The overlapping matches between species are displayed in the Venn diagram. Sorghum unique shows non-homologous gene in an analysis using blastp with an *e*-value ≤0.001 against NCBInr.

Furthermore, these genes were compared with annotated genes from two monocot plants, *Oryza sativa* and *Z. mays* (Phytozome ver. 10). The results are shown in Fig. 3. Using blastn with an *e*-value ≤0.001, 49 (14.6%) and 105 (31.3%) homologous genes were found in *O. sativa* and *Z. mays*, respectively. Because 39 genes were overlapping with both species, 115 newly identified cDNAs were predicted to be homologous to *O. sativa* or *Z. mays*. Of the remaining 221 genes, 186 (55.4%) were non-homologous in an analysis using blastp with an *e*-value ≤0.001 against NCBInr, which suggests that they may be unique sorghum genes.

Natural antisense transcripts control the expression of their sense genes through several mechanisms.^26^ Many paired sense and antisense transcripts have been identified in several plants, such as *Z. mays,*^27^ *O. sativa*^28^ and *Arabidopsis thaliana.*^29,30^ Many of these have been confirmed by means of full-length cDNA mapping onto the genome sequences.^28,31^ Although the tissue-specific expression of non-coding RNA (ncRNA), such as rRNA, tRNA, snoRNA and microRNA, have been analyzed using microarray analysis,^11^ a comprehensive analysis of antisense transcripts in sorghum has not been carried out. In our full-length library, we have identified 323 transcripts from antisense strands of known annotated genes.^18^ To obtain insights into the function of these antisense transcripts, genes on the sense strand corresponding to antisense transcripts were classified based on the GO of molecular function (Supplementary Fig. S2). Using GO Slim, we found that the genes with antisense were widely distributed in the GO categories. The ratios of ‘molecular function’, ‘DNA binding’, ‘transferase activity’ and ‘hydrolase activity’ were higher than all the annotated genes in Sbicolor_255. The results suggest that antisense transcripts play a role in the regulation of sense genes with a wide range of functions.

### 3.2. Expression analysis of newly identified genes and antisense transcripts

We have reported BTx623's transcriptome during the grain's starch-filling phase.^18^ We have prepared RNA from spikelets harvested at the anthesis stage and from seeds harvested 15 days after anthesis. As a vegetative tissue control, we prepared RNA from stems harvested at the same time as the spikelets (Supplementary Fig. S3).^18^

We have generated an integrated transcriptome database to access and use our full-length cDNA data together with our RNA-Seq data and other publicly available data (MOROKOSHI; http://sorghum.riken.jp).^18^

**Figure 4.**
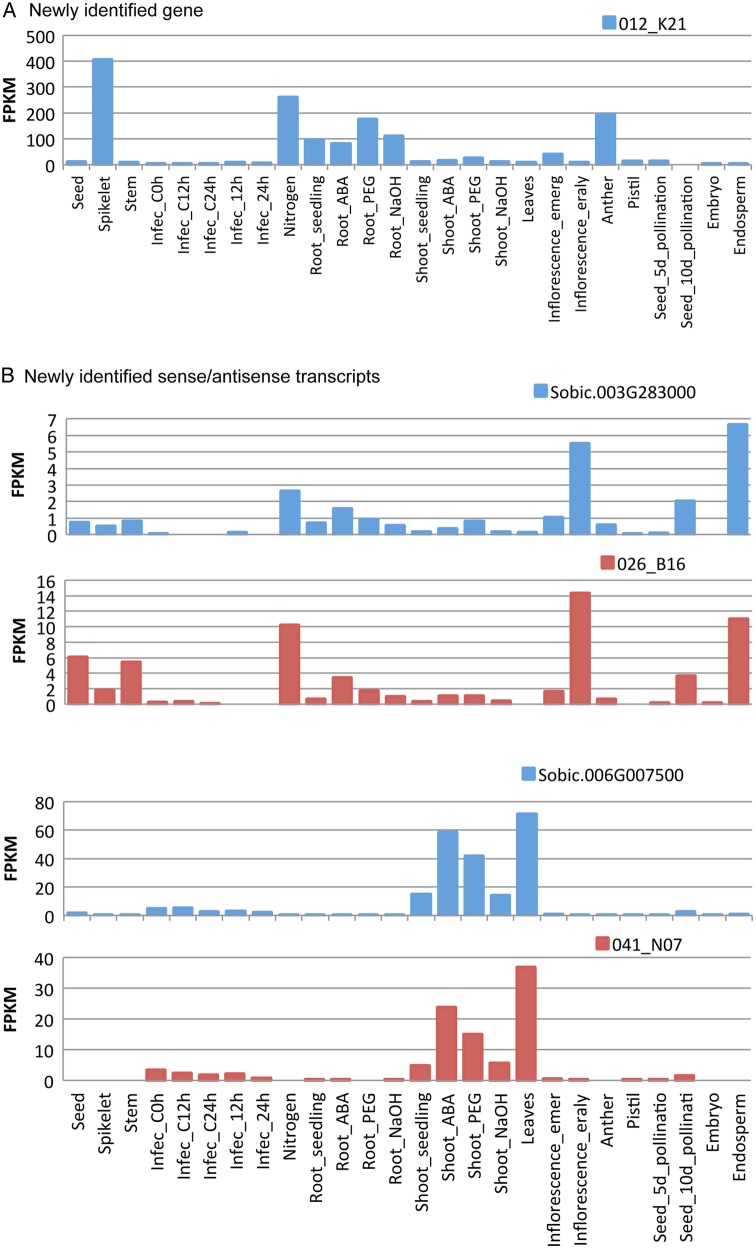
Examples of expression of newly identified gene and sense–antisense transcripts. The plot shows the relative expression (FPKM) of a newly identified gene (A), and sense and antisense transcripts (B) in seed, spikelet and stem from our RNA-Seq data, as well as the publicly available sorghum RNA-Seq data of stressed, infected and tissue-specific samples.^13,15–17^ Blue lines show sense transcripts and red lines show antisense transcripts. This figure is available in black and white in print and in colour at *DNA Research* online.

To obtain insights into the expression of the newly identified genes and the antisense and sense transcripts, the expression patterns were analyzed in our RNA-Seq data as well as the publicly available sorghum RNA-Seq data^13,15–17^ in this study. An example of a newly identified gene is shown in Fig. 4A. The gene, 012_K21, showed spikelet-specific expression in our RNA-Seq data and this result was consistent with the analysis of expression in the anther.^17^ However, it showed expression that was induced by ABA- or PEG stress in the root in other available sorghum RNA-Seq data.^13^ Co-expression analysis using the same method as database, MOROKOSHI^18^ showed some transcription factors (e.g. a Zinc finger-type proteins and homologues of RGL1) co-expressed with this newly identified gene, 012_K21. Examples of newly identified antisense transcripts are shown in Fig. 4B. An identified antisense transcript (026_B16) showed high expression in seed and stem, which is consistent with analysis of expression in the early inflorescence and the endosperm.^17^ In our database, MOROKOSHI,^18^ co-expression analysis showed some transcription factors (e.g. a AP2 domain-containing transcription factor and a myb family transcription factor) co-expressed with this sense transcript (Sobic.003G28300). The effects of antisense transcripts on the expression of sense transcripts are classified into two groups, concordant regulation and discordant regulation.^30^ One of the identified antisense transcripts (041_N07) showed concordant expression with a sense gene (Sobic.006G007500). This gene showed expression in the shoot that was induced by ABA or PEG stress.^13^ The tissue-specific expression of ncRNA, such as rRNA, tRNA, snoRNA and miRNA using microarray analysis has been reported.^11^ In our RNA-Seq data, we detected the expression of 17 ncRNAs (8 miRNAs, 7 tRNAs, 1 rRNA and 1 snoRNA) out of reported 136 ncRNAs in their analysis^11^ (fragments per kilobase of exon per million mapped sequence reads [FPKM] value >0). Expression of these 17 ncRNAs was confirmed on 12 ncRNAs in spikelet, 11 in seed and 13 in stem. Expression of ncRNAs in seed and spikelet suggested that these ncRNAs were activated during the starch-filling phase in grain sorghum.

### 3.3. Analysis of tissue-specific gene expression

We have reported investigating tissue specificity of genes in three samples by using a cut-off FPKM value form only annotated genes.^18^ In our analysis, in order to extract only the DEGs that are specifically expressed from genes already annotated as well as newly identified genes, any having an adjusted *q*-value ≤0.001 against at least one of the other tissues as well as a FPKM value <1 in both other tissues were included. We identified 570 genes that were spikelet specific, 490 genes were seed specific and 390 genes were stem specific. These tissue-specific genes are listed in Supplementary Table S1. The number of stem-specific genes was lower when compared with those that were spikelet or seed specific. From PlnTFDB (http://plntfdb.bio.uni-potsdam.de/v3.0/),^32^ sorghum contains 2312 unique genes of transcription factors. In tissue-specific genes, we found 29 transcription factors in spikelet, 56 transcription factors in seed and 35 transcription factors in stem. This suggested that these many transcription factors contributed the tissue-specific expression. These results suggest that many genes are expressed tissue specifically during the starch-filling phase in grain sorghum.

**Figure 5.**
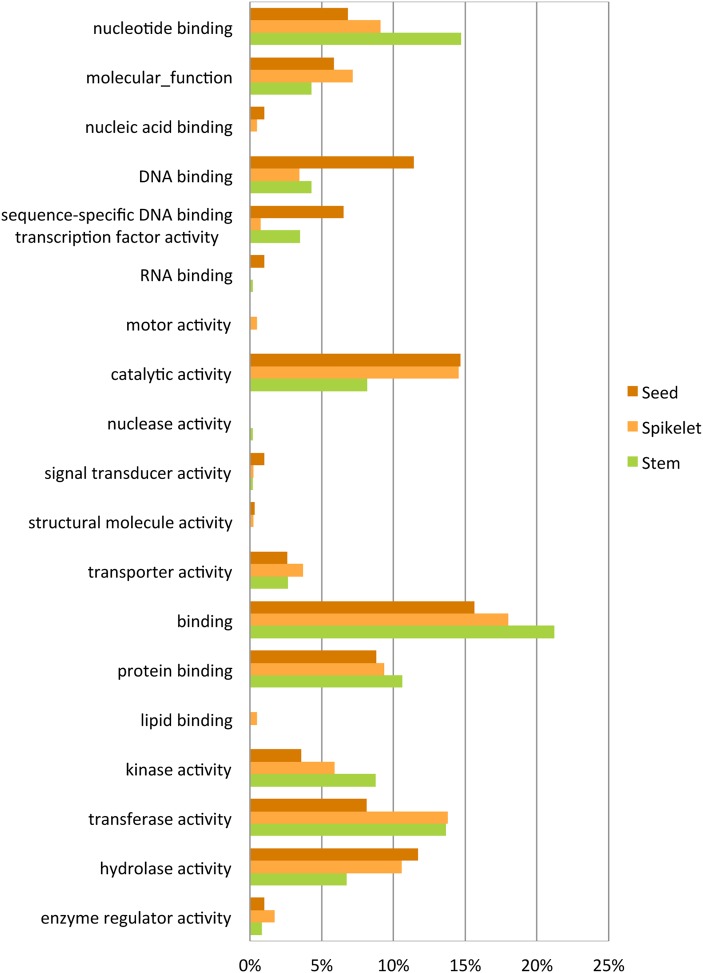
Functional classification based on the GO slim categories of tissue-specific genes. The histogram shows the distribution of genes classified into GO slim categories of specifically expressed genes in each tissue type. Functional annotations are presented in relation to the molecular function.

To gain further insight into the functional significance of tissue-specific genes, we set out to determine whether genes of functional categories were expressed specifically in each tissue. GO functional analysis revealed an enrichment of molecular function in each set consistent with known functional differences (Fig. 5). The distribution of the seed-specific genes set was higher for the categories ‘DNA binding’ and ‘hydrolase activity’ than other tissues. The spikelet-specific genes set was higher for ‘molecular function’, ‘lipid binding’ and ‘enzyme regulator activity’, whereas the stem-specific genes set was higher for ‘nucleotide binding’ and ‘binding’.

In addition to GO analysis, tissue-specific genes were mapped to terms in the KEGG database^22^ to identify significantly enriched metabolic or signal transduction pathways. DEGs between spikelet and stem with KEGG annotation were categorized into 16 pathways, DEGs between seed and stem into 9 pathways and DEGs between seed and spikelet into 16 pathways (Supplementary Table S2). DEGs between spikelet and stem associated with biosynthesis of secondary metabolites, starch and sucrose metabolism, carbon metabolism, plant–pathogen interaction and glycolysis/gluconeogenesis pathways were enriched. DEGs between seed and stem associated with biosynthesis of secondary metabolites, plant hormone signal transduction, starch and sucrose metabolism, biosynthesis of amino acids, 2-oxocarboxylic acid metabolism, and valine, leucine and isoleucine biosynthesis were enriched. Starch and sucrose metabolism pathways were enriched in both DEGs. The carbon metabolism pathway was enriched in only DEGs between spikelet and stem. Plant hormone signal transduction pathways were enriched in only DEGs between seed and stem. This suggests that these metabolic or signal pathways are activated in each tissue.

### 3.4. Analysis of stably expressed genes and non-expressed genes

Stably expressed genes will be useful as controls in future expression experiments. We attempted to find such genes in our RNA-Seq data as well as in publicly available RNA-Seq data.^13,15–17^ We searched for genes with the least variability in expression (coefficient of variation ≤15%) and found 29. They are shown in Supplementary Table S3. Others have identified 448 stably expressed genes.^11^ However, because we used RNA-Seq data from samples that had either been under stress or infected, the number of stably expressed genes was reduced.

Although we analyzed the gene expression of a diverse range of tissues and conditions in our RNA-Seq data as well as in publicly available data, 654 genes of the annotated genes in Sicolor_255 were not detected (FPKM = 0). The list of genes not expressed is shown in Supplementary Table S4. The lack of detection could be due to an absence of expression in vegetative tissues, under the special conditions or at the stages used in this analysis. Other possibilities are that these genes are false positives, or that their levels of expression are below detection limits.

### 3.5. Expression analysis of genes involved in the sucrose-to-starch pathway

**Figure 6.**
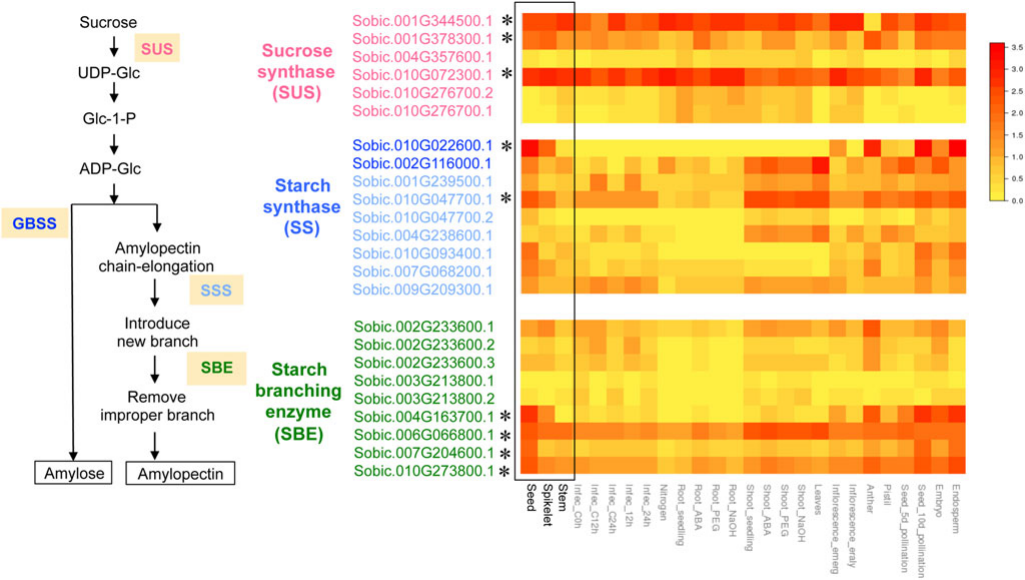
Heat map of tissue-specific expression of genes involved in starch biosynthesis pathway. The heat map shows the relative gene expression (FPKM) in seeds, spikelets and stems from our RNA-Seq data, as well as the publicly available sorghum RNA-Seq data of stressed, infected and tissue-specific samples.^13,15–17^ Asterisks indicate genes that show higher expression in spikelets or seeds.

Grain sorghum has high starch content in its grains and this is the main reason for its cultivation as biofuel feed stock. Datta et al.^33^ examined the expression profiles of some of the genes involved in the sucrose-to-starch pathway at the stage of pollen development. Starch biosynthesis requires the coordination of several enzymes, for which different isozymes have different functions.^34^ However, only limited information is available in the expression profiles of these genes at the stage of seed development. Analysis of expression of isozymes of starch biosynthesis enzymes has been carried out in the genome-wide transcriptome of maize at this stage.^35^ The complete genome sequence of sorghum^2^ and the SorghumCyc, a metabolic pathways database (http://www.gramene.org/pathway/sorghumcyc.html), as well as next-generation sequencing provide a unique opportunity to obtain more precise information about genes and their networks in sorghum. To identify tissue-specific expression patterns, we examined the expression of several known enzymes involved in starch biosynthesis in sorghum. The heat map of expression profiles is shown in Fig. 6. Sucrose synthase (SUS), granule-bound starch synthase (GBSS), soluble starch synthase (SSS) and starch-branching enzyme (SBE) showed 5, 2, 4 and 6 genes, respectively, in the genome (http://www.gramene.org/pathway/sorghumcyc.html). Three of the six *SUS* genes showed high expression in spikelets and seeds. These genes, Sobic.001G344500.1; Sobic.001G378300.1; Sobic.010G072300.1, encode the putative orthologs of maize *Sus1*, *Sus2*, *Shrunken (Sh1),* respectively.^36,37^ The high expression of the ortholog of *Sh1* is consistent with a recent study in maize. *Sh1* mainly accounted for almost all of the transcripts of SUS in the endosperm of maize.^34^ Although expression of *Sus1* and *Sus2* (*SuSxy*) was not detected at the seed development stage in maize, putative orthologs of *Sus1* and *Sus2* were detected in developing seeds in sorghum. A putative ortholog of *Sus2* (Sobic.001G378300.1) showed a specific expression pattern at this stage. There are different types of SS, GBSS and SSS. Two *GBSS* genes showed increased expression at seed development. One gene (Sobic.010G022600.1) showed higher expression than the other. This gene encodes the putative ortholog of maize *waxy1* (*wx1*)*.* This is consistent with maize, in which *waxy1* is mainly expressed in the endosperm.^35^ Of the *SSS* genes, one gene (Sobic.010G047700.1) showed higher expression in seed while another, Sobic.010G047700.2, showed similar expression to the other *SSS* genes. Three of the *SBE* genes, Sobic.004G163700.1, Sobic.007G204600.1 and Sobic.010G273800.1, showed higher expression in seeds and Sobic.006G066800.1 showed expression in spikelets, seeds and stems. Sobic.010G273800.1, Sobic.007G204600.1 and Sobic.004G163700.1 encode orthologs of *amylose-extender1* (*ae1*), *sugary1* (*su1*) and *starch-branching enzyme1* (*sbe1*) of maize, respectively.^38–40^ In Fig. 6, we show expression of stressed, infected and tissue-specific samples from the publicly available sorghum RNA-Seq data.^13,15–17^ One *GBSS* gene (Sobic.002G116000.1) showed higher expression than another (Sobic.010G022600.1) in leaf tissues, although Sobic.002G116000.1 was expressed less in seed tissue. This suggests these genes have separate functions depending on the tissue. These results using publicly available RNA-Seq data indicate that isozymes have different expression patterns in different conditions.

In conclusion, we report here functional analysis of a large-scale collection of full-length cDNAs of *S. bicolor*. Furthermore, we performed genome-wide transcription analysis using RNA-Seq to add information about the expression profiles of our cloned full-length cDNAs. This functional analysis of a large-scale collection of full-length cDNAs with genomic and transcriptome information will facilitate the discovery of functional genes in the Saccharinae and other grass families.

## 4. Data availability

The original cDNA sequence data and the RNA-seq data are available through DDBJ (PRJDB3280 DNA accession) and (PRJDB3281 RNA accession), respectively. The data are also available in MOROKOSHI database (http://sorghum.riken.jp) with several functional annotations.

## Supplementary data

Supplementary data are available at www.dnaresearch.oxfordjournals.org

## Funding

This research was conducted under the research program of the RIKEN Biomass Engineering Program. Funding to pay the Open Access publication charges for this article was provided by RIKEN Biomass Engineering Program.

## Supplementary Material

Supplementary Data
