# Supplementary material for: Functional and expression analyses of transcripts based on full-length cDNAs of Sorghum bicolor
Source: DNA Res. 2015 Nov 5;22(6):485–93. doi: 10.1093/dnares/dsv030 (PMC4675717; doi:10.1093/dnares/dsv030)
Supplement: Supplementary Data [file supp_22_6_485__index.html]

Functional and expression analyses of transcripts based on full-length cDNAs of Sorghum bicolor — Supplementary Data 

# Functional and expression analyses of transcripts based on full-length cDNAs of *Sorghum bicolor*

## Supplementary Data

Supplementary Data

- Supplementary Methods - doc file
- Supplementary Figures - pdf file
- Supplementary Table 1 - xls file
- Supplementary Table 2 - xls file
- Supplementary Table 3 - xls file
- Supplementary Table 4 - xls file
