# Supplementary material for: Functional and expression analyses of transcripts based on full-length cDNAs of Sorghum bicolor
Source: DNA Res. 2015 Nov 5;22(6):485–93. doi: 10.1093/dnares/dsv030 (PMC4675717; doi:10.1093/dnares/dsv030)
Supplement: Supplementary Data [file supp_dsv030_dsv030supp_Methods.doc]

**Supplementary materials and methods**

**Plant material and RNA isolation**

Seeds of sorghum (*Sorghum bicolor)* genotype BTx623 were obtained from the National Institute of Agrobiological Science (NIAS) and used for RNA extraction. Plants were grown in soil in pots in a greenhouse (8-h light: 16-h dark at 30°C). The collection of samples for making the full-length cDNA library and RNA-Seq was described in Makita et al.^1^ The samples for RNA-Seq analysis are shown in Supplementary Fig. S4. RNA extraction was described in Makita et al.^1^

**Construction of full-length cDNA library and Sanger sequencing**

Construction of the full-length cDNA library from poly(A)^+^ RNA was performed by the biotinylated CAP-trapper method, using trehalose-thermoactivated reverse transcriptase.^1,2,3^ The full-length cDNAs were normalized to enrich independent clones and the resulting double-stranded cDNAs were digested with BamHI and XhoI, inserted into the BamHI and SalI sites of a λ-FLC-III vector^4^ and packaged. The original phage library was amplified on solid plates. Subsequently, phage particles were eluted with SM buffer. Plasmids were generated from the amplified phage library by *in vitro* excision. They were transformed into DH10B™ T1 phage-resistant *E. coli* and eluted with SOC medium with 13% glycerol. From this stock, around 40,000 clones were randomly selected, isolated on 384-well plates and cultured in LB medium with 7% glycerol at 30°C, overnight.

Plasmid DNA from each clone was directly amplified from the bacterial cultures in the 384-well plates by the rolling circle amplification (RCA) method using an Illustra TempliPhi DNA Amplification Kit (GE Healthcare, United Kingdom). End sequencing was performed by the Sanger method using ABI 3730xl capillary sequencers (Applied Biosystems, Foster City, CA, USA). The M13Fw (-20) primer (5'-GTAAAACGACGGCCAG-3') and the M13Rvdt primer (5'-GCGGATAACAATTTCACACAGG-3') were used for forward and reverse sequencing, respectively.

**References**

1. Makita, Y., Shimada, S., Kawashima, M., Kondou-kuriyama, T., Toyoda, T., and Matsui, M. 2015, MOROKOSHI : Transcriptome Database in Sorghum bicolor, **56**, 4–11.

2. Carninci, P., Kvam, C., Kitamura, a, et al. 1996, High-efficiency full-length cDNA cloning by biotinylated CAP trapper. *Genomics*, **37**, 327–36.

3. Carninci, P., Nishiyama, Y., Westover, A., et al. 1998, Thermostabilization and thermoactivation of thermolabile enzymes by trehalose and its application for the synthesis of full length cDNA. *Proc. Natl. Acad. Sci. U. S. A.*, **95**, 520–4.

4. Carninci, P., Shibata, Y., Hayatsu, N., et al. 2001, Balanced-size and long-size cloning of full-length, cap-trapped cDNAs into vectors of the novel lambda-FLC family allows enhanced gene discovery rate and functional analysis. *Genomics*, **77**, 79–90.
