# Supplementary material for: Functional and expression analyses of transcripts based on full-length cDNAs of Sorghum bicolor
Source: DNA Res. 2015 Nov 5;22(6):485–93. doi: 10.1093/dnares/dsv030 (PMC4675717; doi:10.1093/dnares/dsv030)
Supplement: Supplementary Data [file supp_dsv030_dsv030supp_figures.pdf]

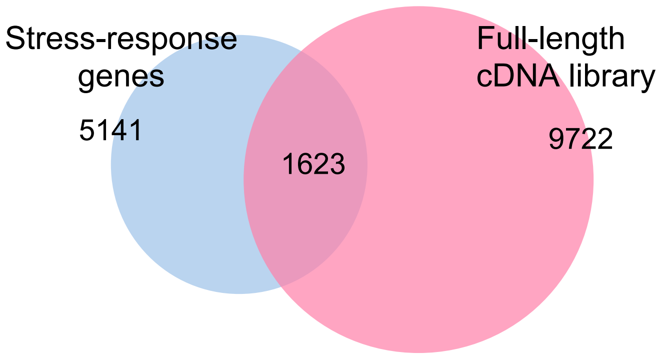

Figure S1. Comparison of genes cloned as full-length cDNAs that are regulated by osmotic stress and abscisic acid treatment. The Venn diagram shows the overlapping matches between stress-response genes regulated by osmotic stress and abscisic acid treatment [Dugas et al. 2011] and genes collected in our full-length cDNA library.

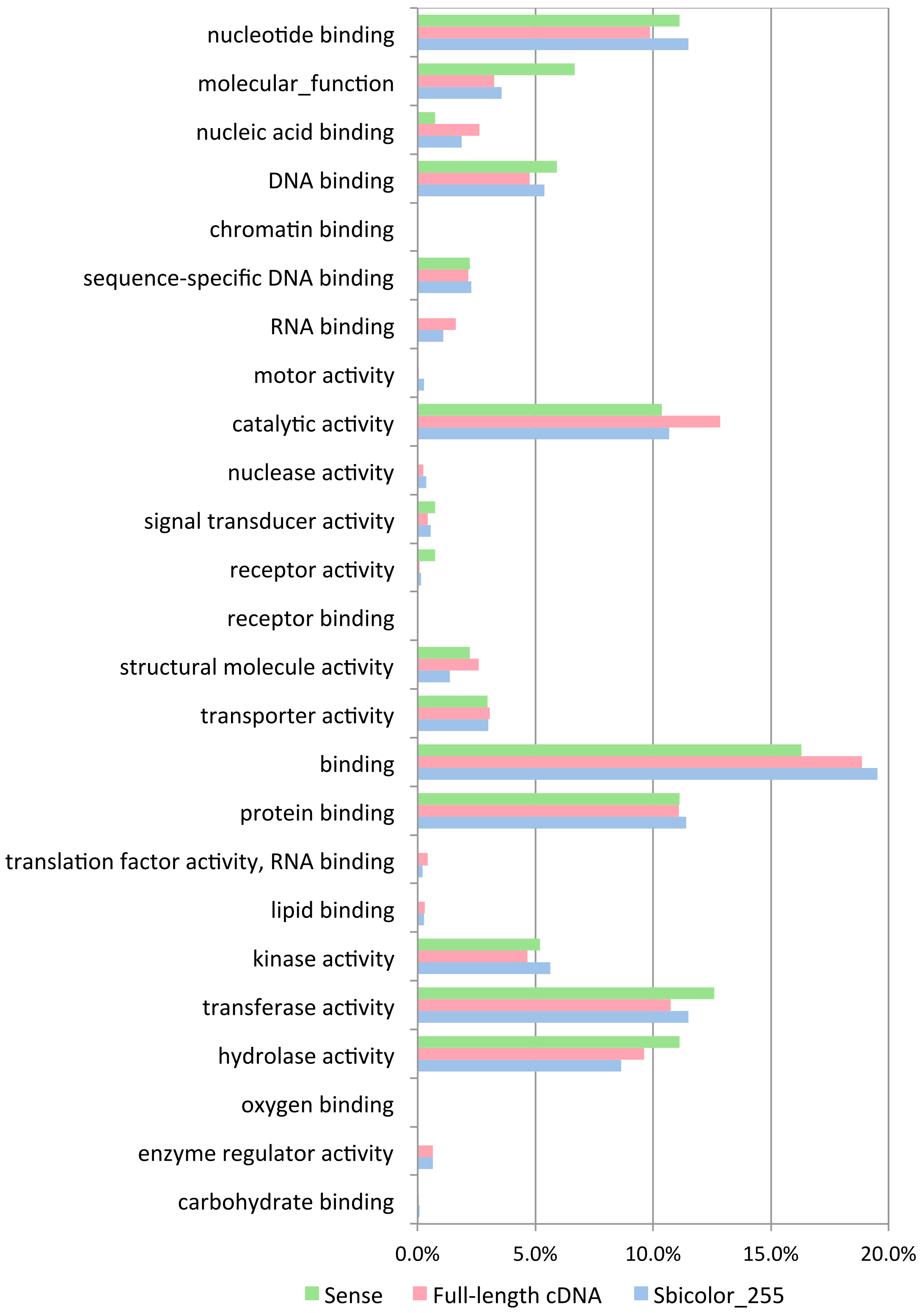

Figure S2. Functional classification based on GO Slim of genes on sense strand corresponding to identified antisense transcripts. The histogram shows the distribution of genes classified into the GO slim categories. Genes were on the sense strand corresponding to identified antisense (green), genes cloned as full-length cDNAs (pink) and total genes annotated in Sbicolor_255 (blue). Functional annotations are presented in relation to the molecular function.

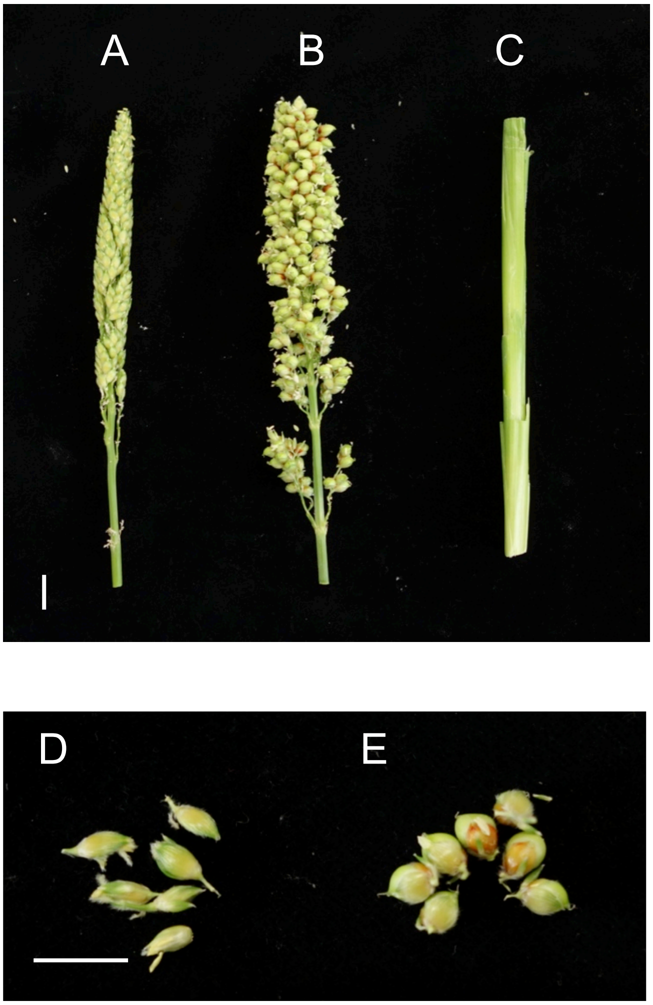

Figure S3. Representative tissues used for RNA-Seq. Tissues collected for preparation of RNA. (A, D) Spikelets harvested at the anthesis stage. (B, E) Seeds harvested 15 days after anthesis. (C) Stems harvested at the same time as spikelets as vegetative tissue controls. Only parts of spikelet (D) and seed (E) were used for RNA extraction. Scale bar = 1 cm.
